# Effectiveness and Safety of Oral Anticoagulants among NVAF Patients with Obesity: Insights from the ARISTOPHANES Study

**DOI:** 10.3390/jcm9061633

**Published:** 2020-05-28

**Authors:** Steve Deitelzweig, Allison Keshishian, Amiee Kang, Amol D. Dhamane, Xuemei Luo, Xiaoyan Li, Neeraja Balachander, Lisa Rosenblatt, Jack Mardekian, Xianying Pan, Manuela Di Fusco, Alessandra B. Garcia Reeves, Huseyin Yuce, Gregory Y. H. Lip

**Affiliations:** 1 Ochsner Clinic Foundation, Department of Hospital Medicine, New Orleans, LA 70115, USA; 2 Ochsner Clinical School, The University of Queensland School of Medicine, New Orleans, LA 70121, USA; 3 STATinMED Research, Ann Arbor, MI 48108, USA; akeshishian@statinmed.com; 4 Bristol-Myers Squibb Company, Lawrenceville, NJ 08648, USA; amiee.kang@bms.com (A.K.); amol.dhamane@bms.com (A.D.D.); shawn.li@bms.com (X.L.); neeraja.balachander@bms.com (N.B.); lisa.rosenblatt@bms.com (L.R.); xianying.pan@bms.com (X.P.); alessandrabg@gmail.com (A.B.G.R.); 5 Pfizer, Inc., Groton, CT 06340, USA; xuemei.luo@pfizer.com; 6 Pfizer, Inc., New York, NY 10017, USA; jmardekian@gmail.com (J.M.); manuela.difusco@pfizer.com (M.D.F.); 7 University of North Carolina, Chapel Hill, NC 27599, USA; 8 New York City College of Technology, City University of New York, New York, NY 11201, USA; hyuce@citytech.cuny.edu; 9 Liverpool Centre for Cardiovascular Science, University of Liverpool and Liverpool Heart & Chest Hospital, Liverpool L69 3BX, UK; gregory.lip@liverpool.ac.uk; 10 Liverpool Heart & Chest Hospital, Liverpool L14 3PE, UK; 11 Aalborg Thrombosis Research Unit, Department of Clinical Medicine, Aalborg University, 9000 Aalborg, Denmark

**Keywords:** stroke, coagulation, outcomes, cardiovascular disease

## Abstract

This ARISTOPHANES analysis examined stroke/systemic embolism (SE) and major bleeding (MB) among a subgroup of nonvalvular atrial fibrillation (NVAF) patients with obesity prescribed warfarin or non-vitamin K antagonist oral anticoagulants (NOACs) in order to inform clinical decision making. A retrospective observational study was conducted among NVAF patients who were obese and initiated apixaban, dabigatran, rivaroxaban, or warfarin from 1 January 2013–30 September 2015, with data pooled from CMS Medicare and four US commercial claims databases. Propensity score matching was completed between NOACs and against warfarin in each database, and the results were pooled. Cox models were used to evaluate the risks of stroke/SE and MB. A total of 88,461 patients with obesity were included in the study. Apixaban and rivaroxaban were associated with a lower risk of stroke/SE vs. warfarin (HR: 0.63, 95% CI: 0.49–0.82 and HR: 0.84, 95% CI: 0.72–0.98). Dabigatran was associated with a similar risk of stroke/SE compared to warfarin. Compared with warfarin, apixaban and dabigatran had a lower risk of MB (HR: 0.54, 95% CI: 0.49–0.61 and HR: 0.75, 95% CI: 0.63–0.91). Rivaroxaban was associated with a similar risk of MB compared to warfarin. In this high-risk population with obesity, NOACs had a varying risk of stroke/SE and MB vs. warfarin.

## 1. Introduction

Atrial fibrillation (AF) is the most common type of arrhythmia in the USA and European countries, with a current estimated prevalence between 1% and 4% [1]. Its prevalence is of critical concern owing to its cardiovascular complications such as ischemic stroke, heart failure, and increasing mortality [2]. Obesity, another prevalent condition worldwide, was estimated to cause 3.4 million deaths in 2010. If current trends continue, forecasts estimate that 1 billion adults will be obese by the year 2030 [3]. Moreover, obesity has been linked with AF, due to its association with obstructive sleep apnea, diabetes mellitus, hypertension, left ventricular dysfunction, heart failure with preserved left ventricular function, and left atrial enlargement [4,5,6]. It has also been associated with hypofibrinolysis, inflammation, and a prothrombotic state, further bolstering the link with the thromboembolic effects of AF [4,5].

In the Atherosclerosis Risk in Communities (ARIC) study, obesity and overweight accounted for 17.9% of all AF cases [6]. Though AF risk appears to follow a linear relationship with an increase in BMI, the pathophysiological basis of the obesity–AF relationship is complex and multifactorial [7]. Exploring the risk of stroke in a subgroup of an AF population with obesity is paramount due to prevalence and the potential for high morbidity and mortality.

In the years since their approval, non-vitamin K oral anticoagulants (NOACs) have been increasingly preferred over warfarin due to the convenience of fewer routine monitoring visits, no requirements for dose adjustment, and limited dietary interactions. With the current fixed-dose NOAC prescriptions, the clinical impact of anticoagulation on non-valvular atrial fibrillation (NVAF) patients with obesity is expected to be similar, provided patients have optimum peak and trough levels of NOACs [8]. The International Society of Thrombosis and Hemostasis recommends the standard dosing of NOACs in patients with obesity and with a BMI ≤ 40 or weight ≤ 120 kg but suggests that NOACs should not be used among patients with a BMI > 40 or weight > 120 kg because there is limited clinical data for these patients [9,10]. The use of NOACs in patients with morbid obesity has not been as well-documented or established. Therefore, comparing the risk of stroke and major bleeding (MB) in a real-world population of NVAF patients with obesity and morbid obesity among oral anticoagulant (OAC) users is crucial.

The NOAC clinical trials RE-LY and ROCKET-AF suggested that there was no significant interaction between weight categories (≥100 kg vs. <100 kg) regarding the impact of dabigatran and rivaroxaban versus warfarin on the risk of stroke/systemic embolism (SE) [11,12]. A similar risk of major and clinically relevant non-MB was also seen among the subgroup with obesity for rivaroxaban vs. warfarin in the ROCKET-AF trial [12]. A post-hoc analysis using the patients in the ARISTOTLE trial showed evidence of significant interaction between BMI and MB, comparing apixaban vs. warfarin, with a larger reduction in MB with normal vs. higher BMI [13].

Additionally, several observational studies evaluating patients with morbid obesity (BMI ≥ 35 or BMI ≥ 40, depending on the source) showed that NOACs had a similar risk of stroke/SE and MB compared to warfarin [14,15,16,17]. While they add valuable knowledge regarding the clinical course of patients with obesity and morbid obesity, existing real-world studies have limitations (e.g., small sample size and no individual NOAC comparisons) that suggest the need for the further evaluation of NOAC treatment in these populations. Larger real-world studies may be warranted to further examine the use of NOACs in this high-risk population with obesity. Using several data sources, this subgroup analysis of ARISTOPHANES (Anticoagulants for Reduction In Stroke: Observational Pooled analysis on Health outcomes And Experience of Patients; NCT03087487) aimed to respectively compare the risk of stroke/SE and MB among the NVAF population with obesity newly prescribed apixaban, dabigatran, rivaroxaban, or warfarin.

## 2. Experimental Section

### 2.1. Data Sources

This study was a retrospective observational database analysis of a patient population of >180 million beneficiaries per year (~56% of the United States population), using fee-for-service (FFS) Medicare data from the US Centers for Medicare & Medicaid Services (CMS) and four US commercial claims databases: the IBM MarketScan^®^ Commercial Claims and Encounter Database, the IQVIA PharMetrics Plus™ Database, the Optum Clinformatics™ Data Mart, and the Humana Research Database. The databases include patients with Medicare FFS, Medicare Advantage, and commercial insurance. Database records include comprehensive demographic and clinical information and International Classification of Diseases, 9th Revision, Clinical Modification (ICD-9-CM) codes, Healthcare Common Procedure Coding System codes, and National Drug Codes. Previously published articles include detailed descriptions of the datasets, the rationale for the pooling process, and the approaches to minimizing potential patient record duplicates across data sources [18,19].

### 2.2. Patient Selection

NVAF patients diagnosed with obesity were selected if they had ≥1 pharmacy claim for apixaban, dabigatran, rivaroxaban, or warfarin between 01 January 2013 and 30 September 2015 (identification period). Edoxaban was excluded from the final sample due to small sample size. Obesity is typically defined as a BMI ≥ 30 kg/m^2^ [20] and was defined here by the presence of a diagnosis code containing obesity or an obese BMI designation (Table A1). The first NOAC prescription date was designated as the index date if patients had a NOAC claim. The first warfarin prescription date was designated as the index date for patients without any NOAC claim. Patients were required to have an AF diagnosis before or on the index date and have continuous medical and pharmacy health plan enrollment for ≥12 months pre-index date (baseline period).

To evaluate new initiators, patients treated with an OAC within 12 months pre-index date were excluded. Patients were also excluded if they had claims indicating any of the following: valvular heart disease, venous thromboembolism, transient AF (pericarditis, hyperthyroidism, or thyrotoxicity), heart valve replacement/transplant, or cardiac surgery during the baseline period; pregnancy during the study period; or hip or knee replacement surgery within 6 weeks pre-index date. In addition, patients were excluded if they had >1 OAC on the index date or had no follow-up. Lastly, patients with claims containing ICD-10 codes were excluded to ensure accurate classification, as the ICD-10-CM coding system was not fully adopted in the United States until 1 October 2015, after the study period ended.

### 2.3. Outcome Measures

The outcome measures were time to first stroke/SE, including ischemic stroke, hemorrhagic stroke, and SE; and time to first MB, including gastrointestinal (GI) bleeding, intracranial hemorrhage, and bleeding at other key sites (e.g., the genitourinary tract, respiratory tract, or ocular area; Table A1) [21,22]. Outcomes were based on hospitalizations with stroke/SE or MB as the principal or first-listed diagnosis. The follow-up period ranged from one day post-index date to 30 days after discontinuation, the switch date, death (only inpatient death for the commercial databases and all-cause death for the Medicare database), the end of continuous medical or pharmacy plan enrollment, or the end of the study (30 September 2015), whichever came first.

### 2.4. Statistical Methods

Propensity score matching (PSM) was conducted between the NOAC and warfarin cohorts (apixaban vs. warfarin, dabigatran vs. warfarin, and rivaroxaban vs. warfarin) and between the NOAC cohorts (apixaban vs. dabigatran, apixaban vs. rivaroxaban, and dabigatran vs. rivaroxaban). Patients were matched 1:1 in each dataset based on the propensity scores generated by logistic regression using demographics, Charlson comorbidity index scores [23], comorbidities, and baseline co-medications. Patients were matched by nearest neighbor matching without replacement (with a caliper of 0.01). Covariate balance was checked through standardized differences, with a threshold of 10% [24]. After ensuring the cohorts were balanced in each database, study patients from the five datasets were pooled for analysis.

The risks of stroke/SE and MB were evaluated using Cox proportional hazard models, with robust sandwich estimates [25]. *p*-values < 0.05 were considered statistically significant. OAC treatment was included as the independent variable; as the cohorts were balanced, no other covariates were included in the model.

### 2.5. Subgroup Analyses

Two subgroup analyses were conducted. First, PSM and Cox proportional hazard models were completed for patients prescribed standard dose NOACs (apixaban 5 mg twice a day (BID), dabigatran 150 mg BID, rivaroxaban 20 mg once a day (QD)). A second subgroup analysis was conducted among patients with morbid obesity. Patients with morbid obesity were defined using diagnosis codes indicating morbid obesity or a BMI ≥ 40 and were re-matched (Table A1) [15]. For both subgroup analyses, the same methodology as that for the main analysis was used.

Institutional Review Board approval was not required because the study did not involve the collection, use, or transmittal of individual identifiable data. Both the datasets and the security of the offices where analysis was completed (and where the datasets are kept) met the requirements of the Health Insurance Portability and Accountability Act of 1996.

## 3. Results

**Figure 1:**
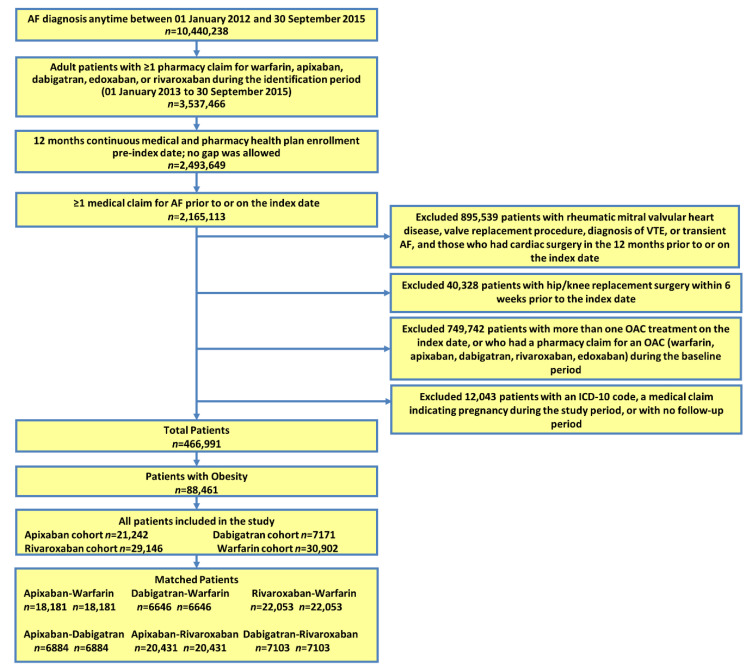
Patient selection figure. AF: atrial fibrillation; OAC: oral anticoagulant; VTE: venous thromboembolism. Edoxaban was excluded from the final population due to a small sample size.

After applying the selection criteria, a total of 88,461 (18.9%) NVAF patients with obesity were identified, including 21,242 apixaban (24.0%), 7171 dabigatran (8.1%), 29,146 rivaroxaban (32.9%), and 30,902 warfarin (34.9%) patients (Figure 1). Before PSM, the warfarin patients were the oldest and had the highest CHA_2_DS_2_-VASc and HAS-BLED scores, followed by apixaban, rivaroxaban, and dabigatran patients (Table A2).

The unadjusted incidence rates of stroke/SE were 2.0, 1.3, 1.5, and 1.3 for warfarin, apixaban, dabigatran, and rivaroxaban per 100 person-years, respectively. The unadjusted rates for MB were 7.6, 3.9, 4.0, and 6.0 per 100 person-years for warfarin, apixaban, dabigatran, and rivaroxaban, respectively (Table A3).

The PSM procedure resulted in 18,181 pairs for the apixaban-warfarin, 6646 pairs for the dabigatran-warfarin, and 22,053 pairs for the rivaroxaban-warfarin cohorts with obesity. Matching for NOAC comparisons included 6884 patient pairs for the apixaban-dabigatran, 20,431 pairs for the apixaban-rivaroxaban, and 7103 pairs for the dabigatran-rivaroxaban cohorts (Figure 1). The mean follow-up time for the six matched cohorts ranged from 6 to 8 months. Within NOAC vs. warfarin comparisons, patients prescribed standard doses of NOACs included 84.8% of apixaban (5 mg), 86.5% of dabigatran (150 mg) and 75.3% of rivaroxaban (20 mg) patients. Select baseline characteristics of the matched populations are shown in Table 1a,b. After matching, all demographic and clinical characteristics were well balanced between pairs (a complete list of baseline variables appears in Table A4 and Table A5).

### 3.1. NOAC vs. Warfarin Comparison

**Figure 2:**
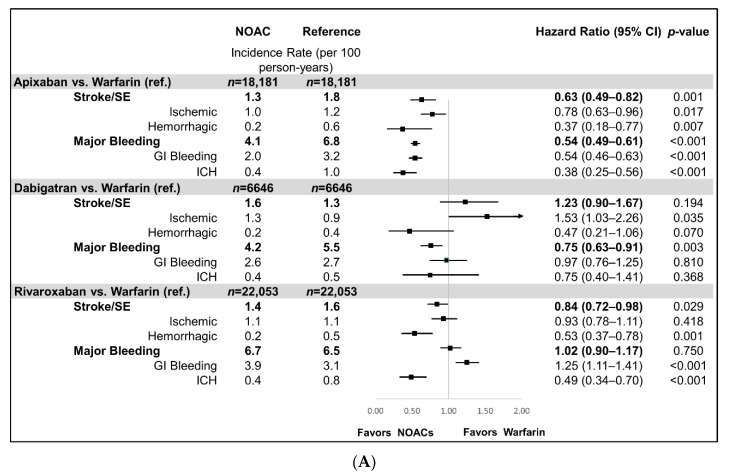

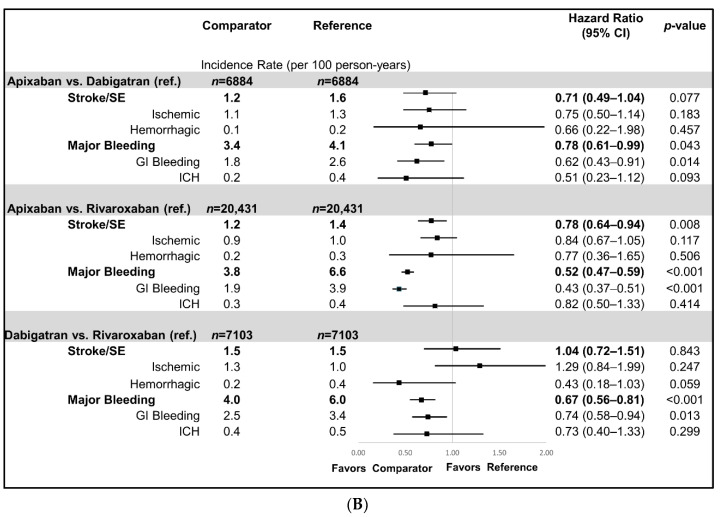
Incidence rates and hazard ratios for stroke/SE and major bleeding among NOACs vs. warfarin and NOACs vs. NOACs. (**A**) NOACs vs. warfarin. (**B**) NOACs vs. NOACs. CI: confidence interval; GI: gastrointestinal; ICH: intracranial hemorrhage; NOACs: non-vitamin K oral anticoagulants; ref: reference; SE: systemic embolism.

Patients prescribed apixaban and rivaroxaban had a lower risk of stroke/SE compared to warfarin patients (apixaban: hazard ratio (HR): 0.63, 95% confidence interval (CI): 0.49–0.82; rivaroxaban: HR: 0.84, 95% CI: 0.72–0.98), while dabigatran patients had a similar risk of stroke/SE compared to warfarin patients (HR: 1.23, 95% CI: 0.90–1.67). Compared with warfarin, apixaban and dabigatran (HR: 0.54, 95% CI: 0.49–0.61; HR: 0.75, 95% CI: 0.63–0.91, respectively) were associated with a lower risk of MB (Figure 2a). Rivaroxaban had a similar risk of MB (HR: 1.02, 95% CI: 0.90–1.17) compared to warfarin.

### 3.2. NOAC vs. NOAC Comparisons

**Figure A1:**
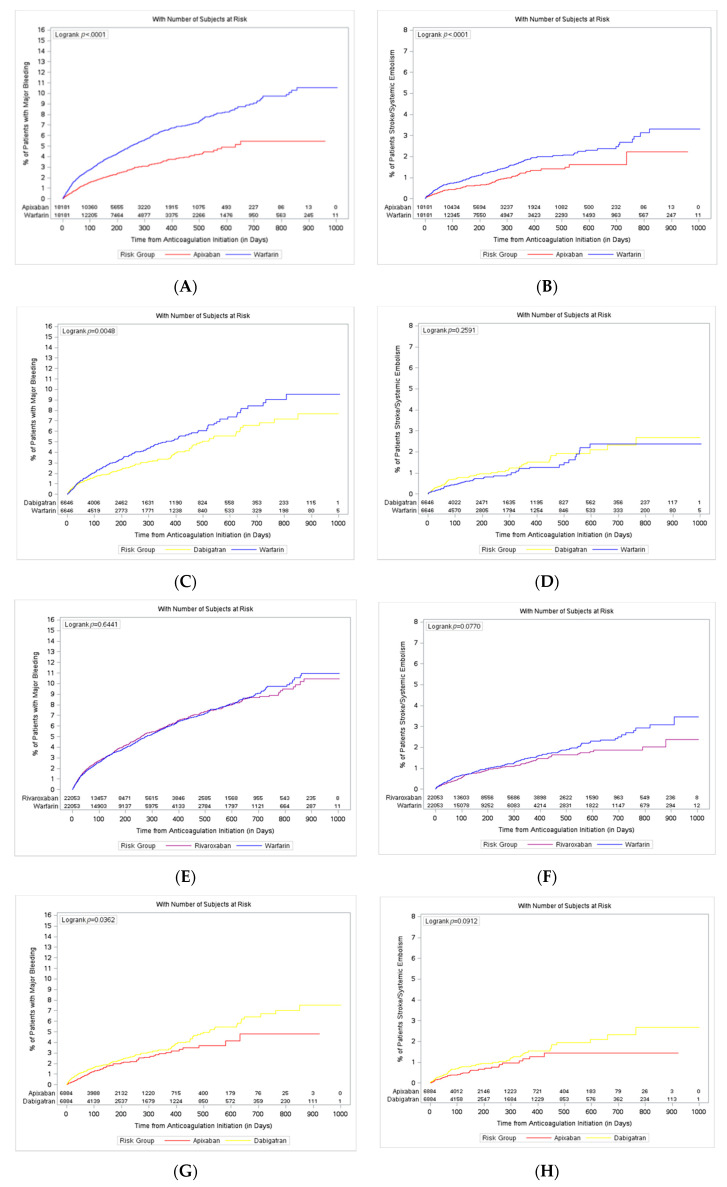

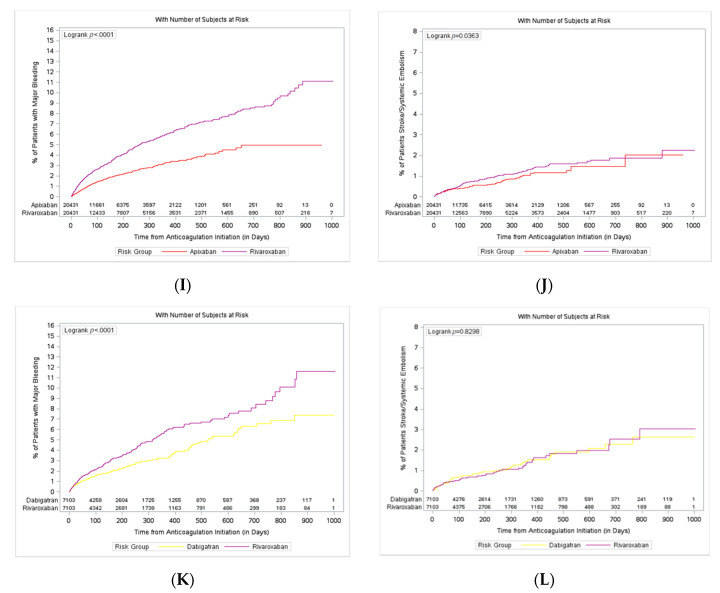
Cumulative incidence of major bleeding and stroke/systemic embolism. (**A**) Major bleeding for apixaban vs. warfarin; (**B**) Stroke/systemic embolism for apixaban vs. warfarin; (**C**) Major bleeding for dabigatran vs. warfarin; (**D**) Stroke/systemic embolism for dabigatran vs. warfarin; (**E**) Major bleeding for rivaroxaban vs. warfarin; (**F**) Stroke/systemic embolism for rivaroxaban vs. warfarin; (**G**) Major bleeding for apixaban vs. dabigatran; (**H**) Stroke/systemic embolism for apixaban vs. dabigatran; (**I**) Major bleeding for apixaban vs. rivaroxaban; (**J**) Stroke/systemic embolism for apixaban vs. rivaroxaban; (**K**) Major bleeding for dabigatran vs. rivaroxaban; (**L**) Stroke/systemic embolism for dabigatran vs. rivaroxaban.

Compared to rivaroxaban, apixaban was associated with a lower risk of stroke/SE (HR: 0.78, 95% CI: 0.64–0.94) and MB (HR: 0.52, 95% CI: 0.47–0.59). Compared to dabigatran, apixaban had a non-significant difference for the risk of stroke/SE (HR: 0.71, 95% CI: 0.49–1.04) and a lower risk of MB (HR: 0.78, 95% CI: 0.61–0.99). Dabigatran was associated with a lower risk of MB (HR: 0.67, 95% CI: 0.56–0.81) than rivaroxaban while having a similar risk of stroke/SE (HR: 1.04, 95% CI: 0.72–1.51) (Figure 2b). The Kaplan–Meier curves for the cumulative incidence rates of stroke/SE and MB in the matched populations have been included in Figure A1(a)–(l)

### 3.3. Subgroup Analyses

The results of the standard dose subgroup analysis were generally consistent with the main analysis. However, there was no significant difference between apixaban and dabigatran for major bleeding (HR: 0.77, 95% CI: 0.59–1.00) and between apixaban and rivaroxaban for stroke/SE (HR: 0.93, 95% CI: 0.74–1.17). (Table 2).

Among all the patients with obesity in the pooled sample, 39.5% were identified as morbidly obese. PSM resulted in 6310 apixaban-warfarin, 2342 dabigatran-warfarin, 8055 rivaroxaban-warfarin, 2373 apixaban-dabigatran, 7180 apixaban-rivaroxaban, and 2617 dabigatran-rivaroxaban pairs of patients. There was no significant difference in the risk of stroke/SE between each NOAC versus warfarin or between NOACs. Apixaban had a lower risk of MB compared to warfarin, dabigatran, and rivaroxaban. Conversely, dabigatran and rivaroxaban were both associated with a similar risk of MB compared to warfarin in the population with morbid obesity (Table 3).

## 4. Discussion

To date, this ARISTOPHANES obesity subgroup analysis is the largest retrospective observational study evaluating the risk of stroke/SE and MB among NVAF patients with obesity who initiated OAC treatment. Due to the increasing prevalence of obesity in the United States, the complexity of case management, and the limited data, we chose to examine the effectiveness and safety of NOACs within an NVAF sub-population with obesity [3,4]. With CMS Medicare and four large US national claims datasets, this study found that NOACs had a varying risk of stroke/SE and MB compared to warfarin and among each other in this population with obesity.

These results are largely consistent with subgroup analysis results from previous randomized controlled trials (RCT)s. Post-hoc obesity analyses from the ARISTOTLE trial demonstrated that BMI (18.5 to 25, 25 to 30, and ≥30) did not have significant interaction with treatment and stroke/SE, death, or MACE (composite of stroke/SE, myocardial infarction, and death) [13]. However, the BMI categories had a significant interaction with MB (P_interaction_ = 0.006); for patients with a BMI ≥ 30, apixaban had a similar risk of MB to warfarin; for patients with normal and overweight BMIs, the risk of MB for apixaban was lower compared to that for warfarin. This trend was also seen in the other BMI categories with varying levels of magnitude. The reason for this is likely multifactorial—it is possible that differences in age and comorbidity levels may confound the risk of bleeding. Sub-analysis from the RE-LY trial examined the effect of dabigatran on the risk of stroke/SE by weight categories (<50 kg, 50–99 kg and ≥100 kg). The interaction of the weight of patients on dabigatran (110 mg and 150 mg) had no significant effect on the risk of stroke/SE (*p* = 0.48 and *p* = 0.42, respectively) [11]. Additionally, sub-analysis from the ROCKET-AF trial showed no significant interaction between the BMI categories and stroke/SE (*p* = 0.537) and MB outcomes (*p* = 0.310), comparing rivaroxaban and warfarin [12]. Therefore, based on weight or BMI, the referenced trials have demonstrated a similar efficacy and risk of safety outcomes between NOACs and warfarin in a population with obesity. Furthermore, in a meta-analysis of RCTs and observational studies among patients with obesity, NOACs showed a similar risk of stroke/SE (HR: 0.84, 95% CI: 0.70–1.03) and MB (HR: 1.03, 95% CI: 0.90–1.18) compared to warfarin [26].

Apart from RCTs, few real-world studies have compared the effectiveness and safety of OACs in an NVAF population with obesity. Additionally, very few studies have compared NOACs individually rather than as a class. In the Dresden NOAC Registry, based in Germany, there was no indication that increased BMI was associated with a lack of NOAC effectiveness or safety [27]. Just as for obesity, very few studies have evaluated the effectiveness of NOACs in a population with morbid obesity. A retrospective cohort study of 64 patients with morbid obesity (BMI > 40) found that NOACs had a similar risk of ischemic stroke/transient ischemic attack and MB compared to warfarin [16]. In a recent real-world analysis, the electronic medical records from patients with morbid obesity (BMI > 40) at Montefiore Medical Center (NY, USA) suggested that apixaban had a similar risk of stroke and bleeding compared to warfarin [14]. Another study from the Montefiore Medical Center found that there was no significant difference in the incidence of stroke or major bleeding between apixaban, rivaroxaban, and warfarin patients [17]. A recent real-world analysis using US Truven MarketScan claims among NVAF patients with morbid obesity found that rivaroxaban showed a similar risk of stroke and MB compared to warfarin [15]. Our analysis on patients with morbid obesity found a similar risk of stroke/SE between NOACs and warfarin and suggested a lower risk of MB with apixaban vs. warfarin, apixaban vs. dabigatran, and apixaban vs. rivaroxaban.

Prior studies have not evaluated the effect of dose among patients with obesity. In our dose subgroup analysis, we found that standard dose apixaban and rivaroxaban were associated with a lower risk of stroke/SE compared to warfarin, and standard-dose apixaban and dabigatran were associated with a lower risk of MB compared to warfarin. In addition to this subgroup analysis, some real-world studies have evaluated the effectiveness of fixed-dose NOACs in patients with obesity [28]. While patients with obesity often require the dose adjustment of drugs due to altered pharmacokinetics, the current recommendations for NOAC therapy imply fixed-dose treatment. Furthermore, it has been found that while the plasma levels of NOACs varied by body weight, the variance was not significant [28]. Further studies are needed to evaluate the use of standard-dose NOACs among patients with obesity.

Compared to previous studies that evaluated the safety and effectiveness of NOACs among NVAF patients with obesity, the ARISTOPHANES pooled study provided a larger sample size with higher statistical power to compare outcomes for each OAC in the NVAF subgroup with obesity. The study findings showed that in this high-risk subgroup of NVAF patients, apixaban and rivaroxaban patients had a lower risk of stroke/SE compared to warfarin patients, and rivaroxaban had a similar risk of MB compared to warfarin. Compared with warfarin, apixaban and dabigatran were associated with a lower risk of MB, while dabigatran patients had a similar risk of stroke/SE. In addition, the study found that compared to dabigatran, apixaban had a non-significant difference for the risk of stroke/SE and had a lower risk of MB. Apixaban patients were also found to have a lower risk of stroke/SE and MB compared to rivaroxaban patients. Dabigatran was associated with a lower risk of MB and a similar risk of stroke/SE when compared to rivaroxaban. These results provide information complementary to the obesity post-hoc and sub-analyses from existing trials. While hypothesis-generating, this real-world evidence supports the fixed dose regimen of NOACs, which appears to maintain safety and effectiveness compared to traditional vitamin K antagonist therapy.

## 5. Limitations

This retrospective observational study has several limitations. First, only statistical associations could be concluded, not causal relationships. Although cohorts were matched through PSM, there were potential residual confounders. This limitation is especially important for interpreting the NOAC vs. NOAC comparison results, which are intended for hypothesis generation, given the lack of head-to-head trials. In addition, since the two drug cohorts were matched independently, conclusions can only be drawn between the matched cohorts, not across the comparisons. Second, due to the nature of the claims studies, outcome measures could only be based on ICD-9-CM codes without further adjustment with precise clinical criteria. More importantly, obesity indicators were ascertained based on ICD-9-CM coding (for ≥30 BMI or an indication of obesity). Body measurements such as weight or lean body mass were not available in the claims data. A separate analysis was conducted to validate the diagnosis codes used to identify obesity and morbid obesity in this study by using one of the five databases linked with electronic medical records [29]. The results from that study showed the obesity diagnosis codes had high positive predictive value (PPV) (89.8%), high specificity (95.2%), and modest sensitivity (48.7%) among newly treated NVAF patients. The morbid obesity diagnosis codes also had high specificity (96.5%) but modest PPV (67.9%) and sensitivity (62.8%) [29]. The modest sensitivity suggests that we may fail to identify some of the patients with obesity and morbid obesity. The moderate PPV for the morbid obesity diagnosis codes indicates that there may be some misclassified patients in this group.

An additional study limitation is that laboratory values—such as international normalized ratio (INR) measurements—are not available in the dataset, so the time in the therapeutic range for the patients prescribed warfarin was indeterminable. Nonetheless, the inclusion of patients with potentially poorer quality control of warfarin therapy in everyday clinical practice may enable the study findings to better reflect real-world situations. Additionally, unobserved heterogeneity may exist across the five data sources. Finally, the results may not reflect the overall NVAF population in the United States because the study did not include uninsured patients and patients solely covered by other public health insurance plans.

## 6. Conclusions

This study, the largest observational study of NVAF patients with obesity, shows that NOACs were associated with a varying risk of stroke/SE and MB compared to warfarin and among each other. Apixaban was associated with a lower risk of stroke/SE and MB compared to warfarin. Additionally, compared with warfarin, dabigatran was associated with a lower risk of MB and a similar risk of stroke/SE; rivaroxaban was associated with a lower risk of stroke/SE and a similar risk of MB. Additional real-world studies are warranted in the population with obesity to understand the impact of NOACs on this high-risk group. These findings may help clinicians better understand the differentiated profile of OACs in an NVAF patient population with obesity.

## Figures and Tables

**Table 1:** Baseline characteristics among NVAF patients with obesity after propensity score matching.

(**A**) Baseline Characteristics among NVAF Patients with Obesity after Propensity Score Matching—NOACs vs. Warfarin.												
	**Apixaban Cohort**		**Warfarin Cohort**		**Dabigatran Cohort**		**Warfarin Cohort**		**Rivaroxaban Cohort**		**Warfarin Cohort**	
	***n*/Mean**	**%/SD**	***n*/Mean**	**%/SD**	***n*/Mean**	**%/SD**	***n*/Mean**	**%/SD**	***n*/Mean**	**%/SD**	***n*/Mean**	**%/SD**
Sample Size	18,181		18,181		6646		6646		22,053		22,053	
Age	72.8	9.0	72.7	8.8	70.7	9.1	70.9	9.3	72.3	8.8	72.3	8.9
Gender												
Male	9260	50.9%	9268	51.0%	3632	54.6%	3633	54.7%	11,313	51.3%	11,372	51.6%
Female	8921	49.1%	8913	49.0%	3014	45.4%	3013	45.3%	10,740	48.7%	10,681	48.4%
Baseline Comorbidity												
Deyo–Charlson Comorbidity Index	3.9	2.9	4.0	2.9	3.5	2.8	3.5	2.9	3.9	2.9	3.9	2.9
CHA_2_DS_2_-VASc Score	4.1	1.6	4.2	1.6	3.8	1.7	3.9	1.6	4.1	1.6	4.1	1.6
HAS-BLED Score ^1^	3.4	1.3	3.4	1.3	3.1	1.3	3.1	1.3	3.3	1.3	3.3	1.3
Dose of the Index Prescription												
Standard Dose ^2^	15,410	84.8%			5747	86.5%			16,599	75.3%		
Low Dose ^3^	2771	15.2%			899	13.5%			5454	24.7%		
Follow-Up Time (in Days)	176.2	160.2	236.3	213.8	222.5	219.7	236.8	211.3	221.0	208.6	237.7	213.5
Median	120		157		128		159		142		159	
(**B**) Baseline Characteristics among NVAF Patients with Obesity after Propensity Score Matching among NOACs vs. NOACs.												
	**Apixaban Cohort**		**Dabigatran Cohort**		**Apixaban Cohort**		**Rivaroxaban Cohort**		**Dabigatran Cohort**		**Rivaroxaban Cohort**	
	***n*/Mean**	**%/SD**	***n*/Mean**	**%/SD**	***n*/Mean**	**%/SD**	***n*/Mean**	**%/SD**	***n*/Mean**	**%/SD**	***n*/Mean**	**%/SD**
Sample Size	6884		6884		20,431		20,431		7103		7103	
Age	70.5	10.0	70.0	9.8	71.5	9.8	71.5	9.7	69.7	10.0	69.5	9.9
Gender												
Male	3776	54.9%	3810	55.3%	10,596	51.9%	10,614	52.0%	3982	56.1%	4171	58.7%
Female	3108	45.1%	3074	44.7%	9835	48.1%	9817	48.0%	3121	43.9%	2932	41.3%
Baseline Comorbidity												
Deyo–Charlson Comorbidity Index	3.3	2.8	3.4	2.8	3.6	2.9	3.6	2.9	3.3	2.8	3.2	2.7
CHA_2_DS_2_-VASc Score	3.7	1.7	3.7	1.7	3.9	1.7	3.9	1.7	3.7	1.7	3.6	1.7
HAS-BLED Score ^1^	3.1	1.4	3.1	1.3	3.2	1.4	3.2	1.4	3.0	1.4	3.0	1.3
Dose of the Index Prescription												
Standard Dose ^2^	6045	87.8%	5979	86.9%	17,634	86.3%	15,514	75.9%	6194	87.2%	5698	80.2%
Low Dose ^3^	839	12.2%	905	13.1%	2797	13.7%	4917	24.1%	909	12.8%	1405	19.8%
Follow-Up Time (in Days)	176.2	158.3	221.5	218.3	176.1	159.6	220.4	208.6	220.7	218.1	217.4	206.4
Median	120		127		120		141		127		140	

CHA_2_DS_2_-VASc: congestive heart failure, hypertension, aged ≥75 years, diabetes mellitus, prior stroke or transient ischemic attack or thromboembolism, vascular disease, aged 65–74 years, sex category; HAS-BLED: hypertension, abnormal renal and liver function, stroke, bleeding, labile international normalized ratios, elderly, drugs and alcohol; NOACs: non-vitamin K oral anticoagulants; NVAF: non-valvular atrial fibrillation; SD: standard deviation. ^1^ As the INR value is not available in the databases, a modified HAS-BLED score was calculated with a range of 0 to 8. ^2^ Standard dose: 5 mg apixaban, 150 mg dabigatran, 20 mg rivaroxaban. ^3^ Lower dose: 2.5 mg apixaban, 75 mg dabigatran, 10 or 15 mg rivaroxaban; (**A**) 1053 patients treated with rivaroxaban were prescribed 10 mg rivaroxaban. (**B**) 950 and 310 patients were prescribed 10 mg of rivaroxaban in the apixaban-rivaroxaban and dabigatran-rivaroxaban cohorts, respectively.

**Table 2:** Incidence and hazard ratios of outcomes for standard-dosed NOACs vs. warfarin and NOACs vs. NOACs.

NOACs vs. Warfarin					NOACs vs. NOACs				
	Incidence per 100 person-years		Hazard Ratio	*p*-value		Incidence per 100 person-years		Hazard Ratio	*p*-value
			(95% CI)					(95% CI)	
	5 mg Apixaban *n* = 15,364	Warfarin *n* = 15,364				5 mg Apixaban *n* = 5904	150 mg Dabigatran *n* = 5904		
Stroke/SE	1.2	1.9	0.61 (0.46–0.81)	0.001	Stroke/SE	1.3	1.3	0.92 (0.55–1.52)	0.733
MB	4.0	6.3	0.57 (0.51–0.64)	<0.001	MB	3.0	3.7	0.77 (0.59–1.00)	0.052
	150 mg Dabigatran *n* = 5756	Warfarin *n* = 5756				5 mg Apixaban *n* = 16,553	20 mg Rivaroxaban *n* = 16,553		
Stroke/SE	1.3	1.2	1.04 (0.70–1.56)	0.843	Stroke/SE	1.2	1.2	0.93 (0.74–1.17)	0.524
MB	3.8	5.0	0.73 (0.56–0.96)	0.024	MB	3.3	5.7	0.53 (0.47–0.60)	<0.001
	20 mg Rivaroxaban *n* = 17,123	Warfarin *n* = 17,123				150 mg Dabigatran *n* = 6169	20 mg Rivaroxaban *n* = 6169		
Stroke/SE	1.2	1.6	0.75 (0.62–0.90)	0.002	Stroke/SE	1.3	1.1	1.18 (0.85–1.65)	0.322
MB	5.8	5.5	1.04 (0.92–1.17)	0.550	MB	3.6	4.9	0.74 (0.61–0.89)	0.002

CI: confidence interval; MB: major bleeding; NOAC: non-vitamin K oral anticoagulants; SE: systemic embolism.

**Table 3:** Incidence rates and hazard ratios of NOACs vs. warfarin and NOACs vs. NOACs among patients with morbid obesity.

NOACs vs. Warfarin					NOACs vs. NOACs				
	Incidence per 100 person-years		Hazard Ratio	*p*-value		Incidence per 100 person-years		Hazard Ratio	*p*-value
			(95% CI)					(95% CI)	
	Apixaban	Warfarin				Apixaban	Dabigatran		
	*n* = 6310	*n* = 6310				*n* = 2373	*n* = 2373		
Stroke/SE	1.3	1.6	0.72	0.113	Stroke/SE	0.7	1.3	0.51	0.175
			(0.48–1.08)					(0.19–1.35)	
MB	4.6	7.8	0.53	<0.001	MB	3.9	5.6	0.63	0.011
			(0.44–0.64)					(0.44–0.90)	
	Dabigatran	Warfarin				Apixaban	Rivaroxaban		
	*n* = 2342	*n* = 2342				*n* = 7180	*n* = 7180		
Stroke/SE	1.5	1.9	0.77	0.304	Stroke/SE	1.3	1.3	0.93	0.618
			(0.47–1.27)					(0.68–1.26)	
MB	5.7	7.4	0.76	0.088	MB	4.0	7.9	0.47	<0.001
			(0.56–1.04)					(0.39–0.56)	
	Rivaroxaban	Warfarin				Dabigatran	Rivaroxaban		
	*n* = 8055	*n* = 8055				*n* = 2617	*n* = 2617		
Stroke/SE	1.3	1.8	0.72	0.079	Stroke/SE	1.4	1.3	1.13	0.629
			(0.50–1.04)					(0.68–1.89)	
MB	8.3	7.6	1.09	0.317	MB	5.4	6.7	0.79	0.086
			(0.92–1.28)					(0.61–1.03)	

CI: confidence interval; MB: major bleeding; NOAC: non-vitamin K oral anticoagulants; SE: systemic embolism.

## Appendix A

**Table A1:** ICD-9-CM diagnosis and procedure codes for selection criteria and outcomes.

Diagnosis	ICD-9-CM Diagnosis and Procedure Codes
Selection Criteria	
Atrial Fibrillation	427.31
Valvular Heart Disease	394.0, 394.1, 394.2, 394.9, 396.0, 396.1, 396.8, 396.9, 424.0, 745.xx
Heart Valve Replacement	V42.2, V43.3, 35.05–35.09, 35.20–35.28, 35.97
VTE	451–453, 671.3, 671.4, 671.9, 415.1, 673.2, 673.8
Transient AF (Heart Valve Replacement/Transplant, Pericarditis, Thyrotoxicity)	Pericarditis: 006.8, 017.9, 036.41, 074.21, 093.81, 098.83, 115.93, 390, 391, 392.0, 393, 411.0, 420.90, 420.91, 420.99, 423.0, 423.1, 423.2, 423.8, 423.9
	Thyrotoxicity: 242.0, 242.1, 242.2, 242.3, 242.4, 242.8, 242.9
Pregnancy	ICD-9-CM: 630–679, V22, V23, V24, V27, V28, V61.6, V61.7, 792.3, 796.5, 72–75.99
	HCPCS: 59000–59350, 76801–76828, 83661–83664
Obesity	278.00 (obesity, unspecified), 278.01 (morbid obesity), 278.03 (obesity hypoventilation syndrome), V85.3 (BMI of 30–39), V85.4 (BMI 40 and over)
Morbid Obesity	278.01 (morbid obesity), V85.4 (BMI 40 and over)
**Outcomes**	
Hemorrhagic Stroke	430.xx–432.xx
	Cases were excluded if traumatic brain injury (ICD-9-CM: 800–804, 850–854) was present during hospitalization.
Ischemic Stroke	433.x1, 434.x1, 436
Systemic Embolism	444.x, 445.x
Major Gastrointestinal Bleeding	456.0, 456.20, 530.82, 531.0x, 531.2x, 531.4x, 531.6x, 532.0x, 532.2x, 532.4x, 532.6x, 533.0x, 533.2x, 533.4x, 533.6x, 534.0x, 534.2x, 534.4x, 534.6x, 535.01, 535.11, 535.21, 535.31, 535.41, 535.51, 535.61, 537.83, 562.02, 562.03, 562.12, 562.13, 568.81, 569.3, 569.85, 578.x
	Procedure code: 44.43
Major Intracranial Hemorrhage	430, 431, 432.0, 432.1, 432.9, 852.0x, 852.2x, 852.4x, 853.0x,
Major Other Hemorrhage	285.1, 360.43, 362.43, 362.81, 363.61, 363.62, 363.72, 364.41, 372.72, 374.81, 376.32, 377.42, 379.23, 423.0x, 596.7x, 599.7x, 602.1x, 620.1, 621.4, 626.2, 626.5, 626.7, 626.8, 626.9, 719.1x, 782.7, 784.7, 784.8, 786.3x, 958.2, 997.02, 998.11
	Procedure code: 99.04

AF: atrial fibrillation; BMI: body mass index; HCPCS: Healthcare Common Procedural Coding System; ICD-9: International Classifications of Diseases, Ninth Edition; VTE: venous thromboembolism.

**Table A2:** Baseline characteristics of NVAF patients with obesity before PSM.

	Warfarin Cohort		Apixaban Cohort		Dabigatran Cohort		Rivaroxaban Cohort	
	*n*/Mean	%/SD	*n*/Mean	%/SD	*n*/Mean	%/SD	*n*/Mean	%/SD
Sample Size	30,902		21,242		7171		29,146	
Age	72.8	8.8	71.5	9.9	69.6	10.0	70.0	10.3
18–54	1038	3.4%	1285	6.0%	606	8.5%	2346	8.0%
55–64	2895	9.4%	2793	13.1%	1112	15.5%	4636	15.9%
65–74	14,044	45.4%	8977	42.3%	3215	44.8%	12,486	42.8%
≥75	12,925	41.8%	8187	38.5%	2238	31.2%	9678	33.2%
Gender								
Male	15,974	51.7%	11,027	51.9%	4033	56.2%	15,647	53.7%
Female	14,928	48.3%	10,215	48.1%	3138	43.8%	13,499	46.3%
U.S. Geographic Region								
Northeast	5330	17.2%	3405	16.0%	1367	19.1%	4944	17.0%
Midwest	9934	32.1%	4980	23.4%	1701	23.7%	7372	25.3%
South	10,693	34.6%	10,103	47.6%	3017	42.1%	12,705	43.6%
West	4857	15.7%	2676	12.6%	1052	14.7%	3988	13.7%
Other	88	0.3%	78	0.4%	34	0.5%	137	0.5%
Race (only for Humana and Medicare)								
White	21,856	88.2%	14,231	89.1%	4376	88.0%	18,059	88.5%
Black	1917	7.7%	1031	6.5%	328	6.6%	1277	6.3%
Other	1004	4.1%	711	4.5%	270	5.4%	1075	5.3%
Baseline Comorbidity								
Deyo–Charlson Comorbidity Index	4.5	3.1	3.7	2.9	3.3	2.8	3.4	2.8
CHADS_2_ Score	2.8	1.3	2.5	1.3	2.4	1.3	2.4	1.3
0	567	1.8%	594	2.8%	262	3.7%	1031	3.5%
1	3942	12.8%	4185	19.7%	1572	21.9%	6374	21.9%
2	8708	28.2%	6475	30.5%	2354	32.8%	9320	32.0%
3+	17,685	57.2%	9988	47.0%	2983	41.6%	12,421	42.6%
CHA_2_DS_2_-VASc Score	4.3	1.6	3.9	1.7	3.7	1.7	3.7	1.7
0	254	0.8%	349	1.6%	169	2.4%	646	2.2%
1	796	2.6%	1045	4.9%	506	7.1%	1936	6.6%
2	2669	8.6%	2842	13.4%	1112	15.5%	4466	15.3%
3	5836	18.9%	4464	21.0%	1610	22.5%	6305	21.6%
4+	21,347	69.1%	12,542	59.0%	3774	52.6%	15,793	54.2%
HAS-BLED Score	3.5	1.4	3.2	1.4	3.0	1.4	3.1	1.4
0	246	0.8%	281	1.3%	133	1.9%	553	1.9%
1	1501	4.9%	1728	8.1%	787	11.0%	2993	10.3%
2	5661	18.3%	4657	21.9%	1793	25.0%	6992	24.0%
3+	23,494	76.0%	14,576	68.6%	4458	62.2%	18,608	63.8%
Bleeding History	8496	27.5%	4425	20.8%	1329	18.5%	5822	20.0%
Congestive Heart Failure	14,722	47.6%	8068	38.0%	2487	34.7%	10,246	35.2%
Diabetes Mellitus	18,984	61.4%	11,390	53.6%	3778	52.7%	15,164	52.0%
Hypertension	29,379	95.1%	20,022	94.3%	6670	93.0%	27,161	93.2%
Renal Disease	12,934	41.9%	6445	30.3%	1720	24.0%	7489	25.7%
Liver Disease	2382	7.7%	1471	6.9%	472	6.6%	2051	7.0%
Myocardial Infarction	4905	15.9%	2528	11.9%	722	10.1%	3235	11.1%
Dyspepsia or Stomach Discomfort	7974	25.8%	5014	23.6%	1555	21.7%	6711	23.0%
Non-Stroke/SE Peripheral Vascular Disease	19,062	61.7%	11,847	55.8%	3745	52.2%	15,274	52.4%
Stroke/SE	4669	15.1%	2307	10.9%	723	10.1%	2937	10.1%
Transient Ischemic Attack	2295	7.4%	1397	6.6%	445	6.2%	1787	6.1%
Anemia and Coagulation Defects	12,178	39.4%	6478	30.5%	1845	25.7%	7949	27.3%
Alcoholism	788	2.5%	517	2.4%	203	2.8%	874	3.0%
Peripheral Artery Disease	8357	27.0%	4563	21.5%	1339	18.7%	6000	20.6%
Coronary Artery Disease	16,865	54.6%	10,616	50.0%	3315	46.2%	13,513	46.4%
Dyslipidemia	25,528	82.6%	17,510	82.4%	5775	80.5%	23,533	80.7%
Morbid Obesity	12,779	41.4%	7962	37.5%	2752	38.4%	11,447	39.3%
Baseline Medication Use								
ACEi/ARB	21,240	68.7%	14,912	70.2%	4964	69.2%	20,093	68.9%
Amiodarone	4143	13.4%	2839	13.4%	859	12.0%	3576	12.3%
Beta Blockers	19,252	62.3%	13,486	63.5%	4347	60.6%	18,192	62.4%
H2-Receptor Antagonist	2663	8.6%	1569	7.4%	485	6.8%	2081	7.1%
Proton Pump Inhibitor	10,973	35.5%	7530	35.4%	2354	32.8%	9922	34.0%
Statins	20,288	65.7%	13,579	63.9%	4324	60.3%	17,928	61.5%
Anti-Platelets	6869	22.2%	4597	21.6%	1244	17.3%	5488	18.8%
NSAIDS	8019	25.9%	6379	30.0%	2194	30.6%	9165	31.4%
Dose of the Index Prescription								
Standard Dose ^1^			18,290	86.1%	6254	87.2%	22,908	78.6%
Lower Dose ^2^			2952	13.9%	917	12.8%	6238	21.4%
Follow-up Time (in Days)	232.4	211.1	175.6	159.1	220.5	217.9	217.9	206.4
Median	154		120		126		139	

ACEi/ARB: angiotensin-converting enzyme inhibitors/angiotensin II receptor blockers; CHA_2_DS_2_-VASc: congestive heart failure, hypertension, aged ≥75 years, diabetes mellitus, prior stroke or transient ischemic attack or thromboembolism, vascular disease, aged 65–74 years, sex category; HAS-BLED: hypertension, abnormal renal and liver function, stroke, bleeding, labile international normalized ratios, elderly, drugs and alcohol; NSAIDs: non-steroidal anti-inflammatory drugs; NVAF: nonvalvular atrial fibrillation; PSM: propensity score matching; SD: standard deviation; SE: systemic embolism. **^1^** Standard dose: 5 mg apixaban, 150 mg dabigatran, 20 mg rivaroxaban. ^2^ Lower dose: 2.5 mg apixaban, 75 mg dabigatran, 10 or 15 mg rivaroxaban; 1311 patients were prescribed 10 mg of rivaroxaban in the rivaroxaban cohort.

**Table A3:** Outcomes characteristics of NVAF patients before PSM.

	Warfarin Cohort		Apixaban Cohort		Dabigatran Cohort		Rivaroxaban Cohort	
	*n*/Mean	%/SD	*n*/Mean	%/SD	*n*/Mean	%/SD	*n*/Mean	%/SD
Sample Size	30,902		21,242		7171		29,146	
Discontinuation	17,553	56.8%	8035	37.8%	4100	57.2%	14,575	50.0%
Time-to-Discontinuation	165.3	163.8	114.9	117.0	141.4	152.7	143.6	153.1
Switch			787	3.7%	748	10.4%	1972	6.8%
Time-to-Switch			94.8	103.0	116.2	136.2	122.7	142.4
Stroke/SE ^1^	406	1.3%	132	0.6%	67	0.9%	226	0.8%
Hemorrhagic Stroke	115	0.4%	23	0.1%			41	0.1%
Ischemic Stroke	276	0.9%	107	0.5%	56	0.8%	170	0.6%
Systemic Embolism	20	0.1%					17	0.1%
Major Bleeding	1491	4.8%	399	1.9%	174	2.4%	1050	3.6%
Gastrointestinal Bleeding	721	2.3%	195	0.9%	110	1.5%	612	2.1%
Intracranial Hemorrhage	190	0.6%	38	0.2%	17	0.2%	67	0.2%
Other Sites	676	2.2%	187	0.9%	55	0.8%	447	1.5%
Stroke/SE Incidence Rate (per 100 person-years)	2.0		1.3		1.5		1.3	
Major Bleeding Incidence Rate (per 100 person-years)	7.6		3.9		4.0		6.0	

NVAF: nonvalvular atrial fibrillation; PSM: propensity score matching; SD: standard deviation; SE: systemic embolism. ^1^ For some cohorts, the number of patients with a stroke event is <11, which cannot be presented per the data use agreement.

**Table A4:** Baseline characteristics among NVAF patients with obesity after propensity score matching—NOACs vs. warfarin.

	Warfarin Cohort		Apixaban Cohort			Warfarin Cohort		Dabigatran Cohort			Warfarin Cohort		Rivaroxaban Cohort		
	*n*/Mean	%/SD	*n*/Mean	%/SD	STD ^1^	*n*/Mean	%/SD	*n*/Mean	%/SD	STD ^1^	*n*/Mean	%/SD	*n*/Mean	%/SD	STD ^1^
Sample Size	18,181		18,181			6646		6646			22,053		22,053		
Age ^2^	72.7	8.8	72.8	9.0	1.52	70.9	9.3	70.7	9.1	1.51	72.3	8.9	72.3	8.8	0.21
18–54	655	3.6%	659	3.6%	0.12	370	5.6%	391	5.9%	1.36	865	3.9%	828	3.8%	0.87
55–64	1746	9.6%	1709	9.4%	0.69	901	13.6%	879	13.2%	0.97	2197	10.0%	2210	10.0%	0.20
65–74	8192	45.1%	8184	45.0%	0.09	3120	46.9%	3147	47.4%	0.81	10,347	46.9%	10,360	47.0%	0.12
≥75	7588	41.7%	7629	42.0%	0.46	2255	33.9%	2229	33.5%	0.83	8644	39.2%	8655	39.2%	0.10
Gender ^2^															
Male	9268	51.0%	9260	50.9%	0.09	3633	54.7%	3632	54.6%	0.03	11,372	51.6%	11,313	51.3%	0.54
Female	8913	49.0%	8921	49.1%	0.09	3013	45.3%	3014	45.4%	0.03	10,681	48.4%	10,740	48.7%	0.54
U.S. Geographic Region ^2^															
Northeast	3132	17.2%	3137	17.3%	0.07	1264	19.0%	1244	18.7%	0.77	3776	17.1%	3828	17.4%	0.62
Midwest	4588	25.2%	4608	25.3%	0.25	1640	24.7%	1624	24.4%	0.56	6275	28.5%	6174	28.0%	1.02
South	7908	43.5%	7834	43.1%	0.82	2643	39.8%	2724	41.0%	2.48	8560	38.8%	8624	39.1%	0.60
West	2501	13.8%	2553	14.0%	0.83	1073	16.1%	1024	15.4%	2.02	3377	15.3%	3365	15.3%	0.15
Other	52	0.3%	49	0.3%	0.31	26	0.4%	30	0.5%	0.93	65	0.3%	62	0.3%	0.25
Race (Only for Humana and Medicare) ^2^															
White	13,165	89.0%	13,190	89.2%	0.54	4367	88.8%	4338	88.2%	1.85	15,805	88.9%	15,796	88.9%	0.16
Black	1010	6.8%	981	6.6%	0.78	298	6.1%	324	6.6%	2.17	1158	6.5%	1178	6.6%	0.45
Other	619	4.2%	623	4.2%	0.13	251	5.1%	254	5.2%	0.28	812	4.6%	801	4.5%	0.30
Baseline Comorbidity															
Deyo–Charlson Comorbidity Index ^2^	4.0	2.9	3.9	2.9	0.92	3.5	2.9	3.5	2.8	3.05	3.9	2.9	3.9	2.9	0.27
CHA_2_DS_2_-VASc Score	4.2	1.6	4.1	1.6	0.61	3.9	1.6	3.8	1.7	1.99	4.1	1.6	4.1	1.6	0.12
0	198	1.1%	165	0.9%	1.83	96	1.4%	107	1.6%	1.35	229	1.0%	222	1.0%	0.32
1	544	3.0%	565	3.1%	0.67	313	4.7%	324	4.9%	0.77	731	3.3%	751	3.4%	0.50
2	1848	10.2%	2029	11.2%	3.23	880	13.2%	951	14.3%	3.10	2306	10.5%	2540	11.5%	3.39
3	3729	20.5%	3718	20.4%	0.15	1559	23.5%	1534	23.1%	0.89	4775	21.7%	4596	20.8%	1.98
4+	11,862	65.2%	11,704	64.4%	1.82	3798	57.1%	3730	56.1%	2.06	14,012	63.5%	13,944	63.2%	0.64
HAS-BLED Score ^3^	3.4	1.3	3.4	1.3	0.54	3.1	1.3	3.1	1.3	1.47	3.3	1.3	3.3	1.3	0.39
0	184	1.0%	144	0.8%	2.33	96	1.4%	93	1.4%	0.38	224	1.0%	198	0.9%	1.21
1	991	5.5%	1063	5.8%	1.72	567	8.5%	565	8.5%	0.11	1289	5.8%	1402	6.4%	2.14
2	3799	20.9%	3696	20.3%	1.40	1585	23.8%	1631	24.5%	1.62	4851	22.0%	4738	21.5%	1.24
3+	13,207	72.6%	13,278	73.0%	0.88	4398	66.2%	4357	65.6%	1.30	15,689	71.1%	15,715	71.3%	0.26
Bleeding History ^2^	4171	22.9%	4127	22.7%	0.58	1346	20.3%	1294	19.5%	1.96	5015	22.7%	5046	22.9%	0.33
Congestive Heart Failure ^2^	7553	41.5%	7485	41.2%	0.76	2436	36.7%	2424	36.5%	0.37	8926	40.5%	8877	40.3%	0.45
Diabetes Mellitus ^2^	10,265	56.5%	10,297	56.6%	0.36	3716	55.9%	3611	54.3%	3.18	12,438	56.4%	12,435	56.4%	0.03
Hypertension ^2^	17,276	95.0%	17,273	95.0%	0.08	6235	93.8%	6223	93.6%	0.74	20,842	94.5%	20,833	94.5%	0.18
Renal Disease ^2^	6173	34.0%	6166	33.9%	0.08	1758	26.5%	1697	25.5%	2.09	6893	31.3%	6891	31.2%	0.02
Liver Disease ^2^	1280	7.0%	1271	7.0%	0.19	472	7.1%	453	6.8%	1.12	1601	7.3%	1620	7.3%	0.33
Myocardial Infarction ^2^	2369	13.0%	2343	12.9%	0.43	700	10.5%	711	10.7%	0.54	2771	12.6%	2794	12.7%	0.31
Dyspepsia or Stomach Discomfort ^2^	4502	24.8%	4430	24.4%	0.92	1486	22.4%	1488	22.4%	0.07	5375	24.4%	5390	24.4%	0.16
Non-Stroke/SE Peripheral Vascular Disease ^2^	10,753	59.1%	10,676	58.7%	0.86	3639	54.8%	3624	54.5%	0.45	12,750	57.8%	12,759	57.9%	0.08
Stroke/SE ^2^	2273	12.5%	2183	12.0%	1.51	739	11.1%	710	10.7%	1.40	2699	12.2%	2639	12.0%	0.83
Transient Ischemic Attack ^2^	1278	7.0%	1257	6.9%	0.45	456	6.9%	432	6.5%	1.45	1511	6.9%	1510	6.8%	0.02
Anemia and Coagulation Defects ^2^	6164	33.9%	6082	33.5%	0.95	1874	28.2%	1814	27.3%	2.02	7167	32.5%	7105	32.2%	0.60
Alcoholism ^2^	428	2.4%	429	2.4%	0.04	192	2.9%	182	2.7%	0.91	621	2.8%	620	2.8%	0.03
Peripheral Artery Disease	4513	24.8%	4228	23.3%	3.67	1464	22.0%	1319	19.8%	5.36	5287	24.0%	5282	24.0%	0.05
Coronary Artery Disease	9491	52.2%	9544	52.5%	0.58	3168	47.7%	3197	48.1%	0.87	11,153	50.6%	11,261	51.1%	0.98
Dyslipidemia ^2^	15,199	83.6%	15,191	83.6%	0.12	5419	81.5%	5402	81.3%	0.66	18,168	82.4%	18,237	82.7%	0.82
Morbid Obesity	7207	39.6%	6777	37.3%	4.86	2619	39.4%	2523	38.0%	2.97	8782	39.8%	8660	39.3%	1.13
Baseline Medication Use ^2^															
ACEi/ARB	12,771	70.2%	12,788	70.3%	0.20	4615	69.4%	4651	70.0%	1.18	15,371	69.7%	15,345	69.6%	0.26
Amiodarone	2429	13.4%	2446	13.5%	0.27	810	12.2%	810	12.2%	0.00	2793	12.7%	2816	12.8%	0.31
Beta Blockers	11,442	62.9%	11,562	63.6%	1.37	4051	61.0%	4025	60.6%	0.80	13,732	62.3%	13,778	62.5%	0.43
H2-Receptor Antagonist	1438	7.9%	1419	7.8%	0.39	500	7.5%	464	7.0%	2.09	1735	7.9%	1760	8.0%	0.42
Proton Pump Inhibitor	6477	35.6%	6510	35.8%	0.38	2259	34.0%	2229	33.5%	0.95	7725	35.0%	7755	35.2%	0.29
Statins	11,915	65.5%	11,913	65.5%	0.02	4213	63.4%	4111	61.9%	3.17	14,141	64.1%	14,210	64.4%	0.65
Anti-Platelets	4070	22.4%	4066	22.4%	0.05	1195	18.0%	1215	18.3%	0.78	4606	20.9%	4585	20.8%	0.23
NSAIDS	5211	28.7%	5255	28.9%	0.53	2025	30.5%	2016	30.3%	0.29	6384	28.9%	6408	29.1%	0.24
Dose of the Index Prescription															
Standard Dose ^4^			15,410	84.8%				5747	86.5%				16,599	75.3%	
Low Dose ^5^			2771	15.2%				899	13.5%				5454	24.7%	
Follow-Up Time (in Days)	236.3	213.8	176.2	160.2	31.85	236.8	211.3	222.5	219.7	6.60	237.7	213.5	221.0	208.6	7.92
Median	157		120			159		128			159		142		

ACEi/ARB: angiotensin converting enzyme inhibitors/angiotensin-receptor blockers; CHA_2_D_S2_-VASc: congestive heart failure, hypertension, aged ≥75 years, diabetes mellitus, prior stroke or transient ischemic attack or thromboembolism, vascular disease, aged 65–74 years, sex category; HAS-BLED: hypertension, abnormal renal and liver function, stroke, bleeding, labile international normalized ratios, elderly, drugs and alcohol; NOACs: non-vitamin K oral anticoagulants; NSAIDs: non-steroidal anti-inflammatory drugs; NVAF: non-valvular atrial fibrillation; SD: standard deviation; SE: systemic embolism; STD: standardized difference. ^1^ Std difference = 100 ×|actual std diff|. Std difference greater than 10 is considered significant. ^2^ Variables used in propensity score matching. ^3^ As the INR value is not available in the databases, a modified HAS-BLED score was calculated with a range of 0 to 8. ^4^ Standard dose: 5 mg apixaban, 150 mg dabigatran, 20 mg rivaroxaban. ^5^ Lower dose: 2.5 mg apixaban, 75 mg dabigatran, 10 or 15 mg rivaroxaban; 1053 patients treated with rivaroxaban were prescribed 10 mg rivaroxaban.

**Table A5:** Baseline characteristics among NVAF patients with obesity after propensity score matching among NOACs vs. NOACs.

	Apixaban Cohort		Dabigatran Cohort			Apixaban Cohort		Rivaroxaban Cohort			Dabigatran Cohort		Rivaroxaban Cohort		
	*n*/Mean	%/SD	*n*/Mean	%/SD	STD ^1^	*n*/Mean	%/SD	*n*/Mean	%/SD	STD ^1^	*n*/Mean	%/SD	*n*/Mean	%/SD	STD ^1^
Sample Size	6884		6884			20,431		20,431			7103		7103		
Age ^2^	70.5	10.0	70.0	9.8	4.23	71.5	9.8	71.5	9.7	0.85	69.7	10.0	69.5	9.9	1.40
18–54	510	7.4%	521	7.6%	0.61	1200	5.9%	1215	5.9%	0.31	590	8.3%	581	8.2%	0.46
55–64	980	14.2%	988	14.4%	0.33	2586	12.7%	2599	12.7%	0.19	1102	15.5%	1102	15.5%	0.00
65–74	3069	44.6%	3159	45.9%	2.63	8828	43.2%	8689	42.5%	1.37	3193	45.0%	3366	47.4%	4.89
≥75	2325	33.8%	2216	32.2%	3.37	7817	38.3%	7928	38.8%	1.12	2218	31.2%	2054	28.9%	5.04
Gender ^2^															
Male	3776	54.9%	3810	55.3%	0.99	10,596	51.9%	10,614	52.0%	0.18	3982	56.1%	4171	58.7%	5.38
Female	3108	45.1%	3074	44.7%	0.99	9835	48.1%	9817	48.0%	0.18	3121	43.9%	2932	41.3%	5.38
U.S. Geographic Region ^2^															
Northeast	1256	18.2%	1255	18.2%	0.04	3339	16.3%	3356	16.4%	0.22	1345	18.9%	1445	20.3%	3.54
Midwest	1624	23.6%	1640	23.8%	0.55	4850	23.7%	4861	23.8%	0.13	1688	23.8%	1613	22.7%	2.50
South	2966	43.1%	2962	43.0%	0.12	9563	46.8%	9580	46.9%	0.17	2999	42.2%	2920	41.1%	2.26
West	1008	14.6%	1000	14.5%	0.33	2606	12.8%	2561	12.5%	0.66	1038	14.6%	1098	15.5%	2.36
Other	30	0.4%	27	0.4%	0.68	73	0.4%	73	0.4%	0.00	33	0.5%	27	0.4%	1.30
Race (Only for Humana and Medicare) ^2^															
White	4361	88.2%	4358	88.1%	0.19	13,910	89.2%	13,926	89.3%	0.33	4368	88.1%	4374	88.2%	0.37
Black	321	6.5%	322	6.5%	0.08	994	6.4%	1001	6.4%	0.18	325	6.6%	318	6.4%	0.57
Other	265	5.4%	267	5.4%	0.18	692	4.4%	669	4.3%	0.72	266	5.4%	267	5.4%	0.09
Baseline Comorbidity															
Deyo–Charlson Comorbidity Index ^2^	3.3	2.8	3.4	2.8	0.27	3.6	2.9	3.6	2.9	0.15	3.3	2.8	3.2	2.7	2.72
CHA_2_DS_2_-VASc Score	3.7	1.7	3.7	1.7	0.57	3.9	1.7	3.9	1.7	0.72	3.7	1.7	3.6	1.7	4.04
0	132	1.9%	142	2.1%	1.04	326	1.6%	337	1.6%	0.43	165	2.3%	167	2.4%	0.19
1	442	6.4%	438	6.4%	0.24	988	4.8%	1025	5.0%	0.84	498	7.0%	516	7.3%	0.98
2	1073	15.6%	1032	15.0%	1.65	2722	13.3%	2642	12.9%	1.16	1100	15.5%	1173	16.5%	2.80
3	1531	22.2%	1547	22.5%	0.56	4326	21.2%	4221	20.7%	1.26	1594	22.4%	1612	22.7%	0.61
4+	3706	53.8%	3725	54.1%	0.55	12,069	59.1%	12,206	59.7%	1.37	3746	52.7%	3635	51.2%	3.13
HAS-BLED Score ^3^	3.1	1.4	3.1	1.3	2.06	3.2	1.4	3.2	1.4	0.10	3.0	1.4	3.0	1.3	4.61
0	96	1.4%	112	1.6%	1.91	266	1.3%	288	1.4%	0.93	130	1.8%	145	2.0%	1.53
1	695	10.1%	680	9.9%	0.73	1640	8.0%	1662	8.1%	0.40	773	10.9%	821	11.6%	2.14
2	1646	23.9%	1708	24.8%	2.10	4508	22.1%	4555	22.3%	0.55	1771	24.9%	1807	25.4%	1.17
3+	4447	64.6%	4384	63.7%	1.91	14,017	68.6%	13,926	68.2%	0.96	4429	62.4%	4330	61.0%	2.87
Bleeding History ^2^	1290	18.7%	1296	18.8%	0.22	4268	20.9%	4265	20.9%	0.04	1317	18.5%	1235	17.4%	3.01
Congestive Heart Failure ^2^	2459	35.7%	2430	35.3%	0.88	7737	37.9%	7779	38.1%	0.42	2471	34.8%	2451	34.5%	0.59
Diabetes Mellitus ^2^	3553	51.6%	3645	52.9%	2.68	10,967	53.7%	11,007	53.9%	0.39	3740	52.7%	3773	53.1%	0.93
Hypertension ^2^	6418	93.2%	6435	93.5%	0.99	19,262	94.3%	19,266	94.3%	0.08	6611	93.1%	6569	92.5%	2.28
Renal Disease ^2^	1728	25.1%	1690	24.5%	1.28	6072	29.7%	6109	29.9%	0.40	1708	24.0%	1580	22.2%	4.27
Liver Disease ^2^	448	6.5%	466	6.8%	1.05	1423	7.0%	1379	6.7%	0.85	471	6.6%	474	6.7%	0.17
Myocardial Infarction ^2^	711	10.3%	706	10.3%	0.24	2400	11.7%	2426	11.9%	0.39	716	10.1%	692	9.7%	1.13
Dyspepsia or Stomach Discomfort ^2^	1514	22.0%	1505	21.9%	0.32	4826	23.6%	4768	23.3%	0.67	1544	21.7%	1478	20.8%	2.27
Non-Stroke/SE Peripheral Vascular Disease ^2^	3676	53.4%	3648	53.0%	0.82	11,365	55.6%	11,484	56.2%	1.17	3712	52.3%	3710	52.2%	0.06
Stroke/SE ^2^	720	10.5%	704	10.2%	0.76	2194	10.7%	2192	10.7%	0.03	711	10.0%	733	10.3%	1.02
Transient Ischemic Attack ^2^	414	6.0%	427	6.2%	0.79	1316	6.4%	1339	6.6%	0.46	442	6.2%	444	6.3%	0.12
Anemia and Coagulation Defects ^2^	1873	27.2%	1815	26.4%	1.90	6197	30.3%	6242	30.6%	0.48	1838	25.9%	1709	24.1%	4.20
Alcoholism ^2^	186	2.7%	195	2.8%	0.80	483	2.4%	471	2.3%	0.39	203	2.9%	221	3.1%	1.49
Peripheral Artery Disease	1350	19.6%	1311	19.0%	1.43	4364	21.4%	4677	22.9%	3.69	1327	18.7%	1463	20.6%	4.82
Coronary Artery Disease	3281	47.7%	3230	46.9%	1.48	10,183	49.8%	10,138	49.6%	0.44	3285	46.2%	3222	45.4%	1.78
Dyslipidemia ^2^	5560	80.8%	5568	80.9%	0.30	16,857	82.5%	16,842	82.4%	0.19	5716	80.5%	5688	80.1%	0.99
Morbid Obesity	2601	37.8%	2658	38.6%	1.70	7660	37.5%	7896	38.6%	2.38	2726	38.4%	2735	38.5%	0.26
Baseline Medication Use ^2^															
ACEi/ARB	4805	69.8%	4799	69.7%	0.19	14,341	70.2%	14,312	70.1%	0.31	4922	69.3%	4929	69.4%	0.21
Amiodarone	827	12.0%	836	12.1%	0.40	2671	13.1%	2658	13.0%	0.19	849	12.0%	874	12.3%	1.08
Beta Blockers	4287	62.3%	4198	61.0%	2.66	12,981	63.5%	12,917	63.2%	0.65	4311	60.7%	4260	60.0%	1.47
H2-Receptor Antagonist	493	7.2%	477	6.9%	0.91	1496	7.3%	1473	7.2%	0.43	482	6.8%	460	6.5%	1.24
Proton Pump Inhibitor	2301	33.4%	2275	33.0%	0.80	7222	35.3%	7151	35.0%	0.73	2342	33.0%	2259	31.8%	2.50
Statins	4212	61.2%	4193	60.9%	0.57	13,057	63.9%	13,149	64.4%	0.94	4285	60.3%	4189	59.0%	2.75
Anti-Platelets	1312	19.1%	1221	17.7%	3.41	4328	21.2%	4392	21.5%	0.76	1235	17.4%	1150	16.2%	3.20
NSAIDS	2119	30.8%	2122	30.8%	0.09	6191	30.3%	6230	30.5%	0.41	2183	30.7%	2104	29.6%	2.42
Dose of the Index Prescription															
Standard Dose ^4^	6045	87.8%	5979	86.9%	2.88	17,634	86.3%	15,514	75.9%	26.75	6194	87.2%	5698	80.2%	18.99
Low Dose ^5^	839	12.2%	905	13.1%	2.88	2797	13.7%	4917	24.1%	15.45	909	12.8%	1405	19.8%	7.53
Follow-Up Time (in Days)	176.2	158.3	221.5	218.3	23.72	176.1	159.6	220.4	208.6	23.89	220.7	218.1	217.4	206.4	1.54
Median	120		127			120		141			127		140		

ACEi/ARB: angiotensin converting enzyme inhibitors/angiotensin-receptor blockers; CHA_2_DS_2_-VASc: congestive heart failure, hypertension, aged ≥ 75 years, diabetes mellitus, prior stroke or transient ischemic attack or thromboembolism, vascular disease, aged 65–74 years, sex category; HAS-BLED: hypertension, abnormal renal and liver function, stroke, bleeding, labile international normalized ratios, elderly, drugs and alcohol; NOACs: non-vitamin K oral anticoagulants; NSAIDs: non-steroidal anti-inflammatory drugs; NVAF: non-valvular atrial fibrillation; PSM: propensity score matching; SD: standard deviation; SE: systemic embolism; STD: standardized difference. ^1^ Std difference = 100 ×|actual std diff|. Std difference greater than 10 is considered significant. ^2^ Variables used in propensity score matching. ^3^ As the INR value is not available in the databases, a modified HAS-BLED score was calculated with a range of 0 to 8. ^4^ Standard dose: 5 mg apixaban, 150 mg dabigatran, 20 mg rivaroxaban. ^5^ Lower dose: 2.5 mg apixaban, 75 mg dabigatran, 10 or 15 mg rivaroxaban; 950 and 310 patients were prescribed 10 mg of rivaroxaban in the apixaban-rivaroxaban and dabigatran-rivaroxaban cohorts, respectively.
